# MicroRNA let-7a suppresses breast cancer cell migration and invasion through downregulation of C-C chemokine receptor type 7

**DOI:** 10.1186/bcr3098

**Published:** 2012-01-18

**Authors:** Seok-Jun Kim, Ji-Young Shin, Kang-Duck Lee, Young-Ki Bae, Ki Woong Sung, Seok Jin Nam, Kyung-Hee Chun

**Affiliations:** 1 Gastric Cancer Branch, Division of Translational & Clinical Research I, National Cancer Center, 323 Ilsan-ro, Ilsandong-gu, Goyang-si, Gyeonggi-do 410-769, Republic of Korea; 2 Cancer Experimental Recourses Branch, Division of Cancer Biology, National Cancer Center, 323 Ilsan-ro, Ilsandong-gu, Goyang-si, Gyeonggi-do 410-769, Republic of Korea; 3 Department of Surgery, School of Medicine, Sungkyunkwan University, Samsung Medical Center, 50 Irwon-dong, Gangnam-gu, Seoul 135-710, Republic of Korea; 4 Department of Pediatrics, School of Medicine, Sungkyunkwan University, Samsung Medical Center, 50 Irwon-dong, Gangnam-gu, Seoul 135-710, Republic of Korea; 5 Department of Biochemistry and Molecular Biology, Yonsei University College of Medicine 250 Sungsan-ro, Seodaemun-gu, Seoul 120-752, Korea

## Abstract

**Introduction:**

C-C chemokine receptor type 7 (CCR7) plays an important role in chemotactic and metastatic responses in various cancers, including breast cancer. In the present study, the authors demonstrated that microRNA (miRNA) let-7a downregulates CCR7 expression and directly influences the migration and invasion of breast cancer cells.

**Methods:**

The expression of CCR7, its ligand CCL21, and let-7a was detected in breast cancer cell lines and in breast cancer patient tissues. Synthetic let-7a and an inhibitor of let-7a were transfected into MDA-MB-231 and MCF-7 breast cancer cells, respectively, and cell proliferation, cell migration, and invasion assays were performed. To confirm the fact that 3'UTR of CCR7 is a direct target of let-7a, a luciferase assay for the reporter gene expressing the let-7a binding sites of CCR7 3'UTR was used. An *in vivo *invasion animal model system using transparent zebrafish embryos was also established to determine the let-7a effect on breast cancer cell invasion.

**Results:**

First, a higher expression of both CCR7 and CCL21 in malignant tissues than in their normal counterparts from breast cancer patients was observed. In addition, a reverse correlation in the expression of CCR7 and let-7a in breast cancer cell lines and breast cancer patient tissues was detected. Synthetic let-7a decreased breast cancer cell proliferation, migration, and invasion, as well as CCR7 protein expression in MDA-MB-231 cells. The let-7a inhibitor reversed the let-7a effects on the MCF-7 cells. The 3'UTR of CCR7 was confirmed as a direct target of let-7a by using the luciferase assay for the reporter gene expressing let-7a CCR7 3'UTR binding sites. Notably, when analyzing *in vivo *invasion, MDA-MB 231 cells after synthetic let-7a transfection were unable to invade the vessels in zebrafish embryos.

**Conclusions:**

The results from the present study suggest that targeting of CCL21-CCR7 signaling is a valid approach for breast cancer therapy and that let-7a directly binds to the 3'UTR of CCR7 and blocks its protein expression, thereby suppressing migration and invasion of human breast cancer cells. Furthermore, the present study underscores the therapeutic potential of let-7a as an antitumor and antimetastatic manager in breast cancer patients.

## Introduction

Breast cancer is the most common cancer in women and is ranked second only to lung cancer in deaths caused by cancer [1]. Although many advanced treatments have resulted from improving clinical instruments and methods, metastasis still leads to cancer mortality and poor prognosis [2]. Therefore, inhibition of metastasis can be a major goal, and we noted that C-C chemokine receptor type 7 (CCR7) is a reasonable therapeutic target in breast cancer therapy, because expression of CCR7 is reportedly correlated with lymphatic metastasis and poor prognosis in breast cancers [3-5]. CCR7 is a member of the G protein-coupled receptor **(**GPCR) family and is commonly expressed on memory T cells, B cells, and mature dendritic cells [4,6]. CCR7 can be activated by the binding of cytokines CCL19 and CCL21 [5]. Usually, naïve T cells enter lymphoid tissues from the blood via its ligands, CCL19 and CCL21, which are expressed by mature dendritic cells [7]. Consequently, these ligands are important for the adaptive immune responses between dendritic cells, B cells, T cells, and the inflammatory response. Therefore, a possible role of CCR7 in cancer development and metastasis is its association with cancers, as well as being linked to the expression of its ligands, CCL19 and CCL21 [8]. Recently, CCL21 was shown to be secreted not only by lymphatic endothelial cells (LECs), but also by the cancer cells themselves [9]. Supposedly, a ligand of CCR7 is secreted by the cancer cells themselves and allows the cancer cells to migrate to the lymphoid tissues; thus, blocking CCR7 in cancer cells could inhibit metastasis without the immune cell responses.

Therefore, we studied the inhibition of CCR7 expression in breast cancer cells. Recently, numerous articles demonstrated that microRNA (miRNA) suppresses the expression of several cancer-related genes and reduces tumorigenesis and metastasis in breast and several other cancers. Specifically, the relative roles of CCR7 and miRNA in breast cancer metastasis were examined, and thus, the CCR7 3'UTR binding miRNA was searched by using the TargetScan prediction program. The results showed that only the let-7 family binds CCR7 3'UTR. Let-7 was the first identified miRNA originally isolated from *Caenorhabditis elegans*. The let-7 family has multiple functions. Within the let-7 family, let-7a expression increases after differentiation and in mature tissue, but is barely detectable in the embryonic stage [10]. Let-7a was found to act as a tumor suppressor directly regulating RAS and HMGA2 oncogenes by interacting with the 3'UTR [11-13]. Reduced levels of let-7a correlate with elevated RAS expression in lung squamous carcinoma [11]. Let-7a is involved in cell proliferation [14] and influences cancer metastasis in various tumors, including breast cancer [15-17]. However, the mechanism of let-7a action on metastasis-related genes is poorly understood.

In the present study, among the metastasis-related genes as potential targets of let-7a, a reverse correlation between CCR7 and let-7a expression was observed in breast cancer patient tissues and breast cancer cell lines, and let-7a specifically influenced CCR7 downexpression. Let-7a was also found to target CCR7 3'UTR directly, thereby downregulating breast cancer cell migration and invasion. Last, by using zebrafish embryo models, confirmation of the let-7a effects observed *in vitro *was obtained *in vivo*. Through the previously mentioned studies, we focused on the interrelation of the two agents, let-7a and CCR7, in search of promising molecular targets to inhibit metastasis and for potential antimetastatic agents for possible use in breast cancer therapy.

## Materials and methods

### Tissues

Tissue samples were obtained at biopsy from 15 breast cancer patients (five infiltrating ductal cancer, one metaplastic cancer matrix-producing type, seven invasive ductal cancer, one infiltrating cribriform cancer, and one atypical medullary cancer) and their normal counterparts at Samsung Medical Center in Korea. The protocol for the study was approved by the institutional review board (IRB) at Samsung Medical Center (1). All participants provided written informed consent and the use of tissues for comprehensive experiments of breast cancer informed consent. Immediately after biopsy, the tissue samples were frozen in liquid nitrogen and stored at -70°C until use.

### Cell culture and transfection

Human breast cancer cell lines, SK-BR-3, MDA-MB-231, MCF-7, Hs-578T, T47D, ZR-75-1, and JIMT-1 were obtained from the ATCC and maintained in RPMI 1640 and Dulbecco modified Eagle medium (DMEM) containing 10% fetal bovine serum (FBS) and 1% antibiotics (Invitrogen, San Diego, CA, USA). The dsRNA used in transfection experiments as a scrambled siRNA (scRNA) was 5'-UCACAACCUCCUAGAAAGAGUAGA-3', synthetic let-7a: 5'-UGAGGUAGUAGGUUGUAUAGUU-3', CCL21 siRNA: 5'- GUACAGCCAAAGGAAGAUUUU-3', and CCR7 siRNA: 5'- GCTGGTCGTGTTGA CCTAT-3'. All of these were synthesized by Genolution Company in Korea. In addition, commercial anti-let-7a oligonucleotide was purchased from Panagene in Korea. The dsRNA was transfected into MDA-MB-231 and MCF-7 cell lines by using Lipofectamine RNAiMAX and Lipofectamine 2000 reagents (Invitrogen), according to the reagent manufacturer's instructions. The MDA-MB-231 and MCF-7 cell lines were harvested 2 days after transfection, and various analyses were performed.

### RNA isolation and reverse transcription polymerase chain reaction

Total RNA was isolated from seven breast cancer cell lines and 10 breast cancer patients' tissues, respectively, by using TRIzol reagent (Invitrogen) and following the manufacturer's instructions. Reverse transcription polymerase chain reaction (RT-PCR) was performed by using the Reverse Transcription System (Promega, Madison, WI, USA). PCR experiments were performed after reverse transcription. The primers for β-actin were 5'-AGCCTCGCCTTTGCCGA-3' and 5'-CTGGTGCCTGGGGCG-3'; for CCR7, 5'-GATGCGATGCTCTCTCATCA-3' and 5'-TGTAGGGCAGCTGGAAGACT-3', and for CCL21, 5'-GCCTTGCCACACTCTTTCTC-3' and 5'-CAAGGAAGAGGTGGGGTGTA-3'. The PCR was performed by using Ex-Taq (TaKaRa) methods.

### microRNA real-time RT-PCR

A TaqMan One-step RT-PCR Master Mix Reagents kit, purchased from Applied Biosystems (ABI, Foster City, CA, USA) was used. Amplification and detection were performed by using a 7900HT Sequence Detection System (ABI) with 40 cycles of denaturation at 95°C (15 seconds) and annealing/extension at 60°C (60 seconds). This was preceded by reverse transcription at 50°C for 30 minutes and denaturation at 95°C for 10 minutes. To quantitate mature miRNA, TaqMan MicroRNA Assays kits were purchased from ABI to detect let-7a and a control miRNA (RNU6B) (ABI). The protocol is two steps, requiring reverse transcription with an miRNA-specific primer, followed by real-time PCR with TaqMan probes (ABI).

### Western blot analysis

Protein was extracted from seven breast cancer cell lines and 10 breast cancer patient tissue samples by using RIPA buffer (Biosesang), including a protease inhibitor cocktail (Sigma) and TRIzol reagent (Invitrogen), according to the manufacturers' instructions. Next, Western blotting was performed by using anti-β-actin, anti-CKR7 (CCR7), anti-IGF-1R (Santa Cruz) anti-c-Myc, and anti-CDK4 (Cell Signaling) antibodies. The signals were detected with an ECL kit (Amersham) by following the manufacturer's instructions.

### Trans-filter migration and invasion assays

Trans-filter migration and invasion assays were performed on MDA-MB-231 and MCF-7 cell lines in serum-free DMEM with 8.0-μm pore inserts on a 24-well Transwell (Corning Costar, Lowell, MA, USA). The cell lines were transfected with synthetic let-7a, CCR7 siRNA, anti-let-7a, and scRNA for 1 day and then transferred to the upper chamber of the Transwell coated with 0.5 mg/ml collagen type I (BD Bioscience) and a 1:15 dilution of Matrigel (BD Bioscience). Migrating and invading cells were quantified after H&E staining. Migration and invasion assays were performed after transfection, as previously described [18].

### The luciferase reporter plasmid constructions

The luciferase reporter constructs were generated by introducing the CCR7 3'UTR carrying a let-7a binding site into the pGL3 control vector (Promega). First, the CCR7 3'UTR wild-type (WT) sequence was amplified with PCR by using primers WT-FW (including *Xba *I restriction enzyme sites) 5'-CTAGTCTAGAGCGACTCTTCTGCCTGG-3' and WT-BW 5'- CTAGTCTAGAGCCATTTACCAAAGACTTTTTTTTTC-3' with MDA-MB-231 cDNA as a template, followed by PCR for the CCR7 3'UTR mutant type (MUT) sequence by using primers MUT-FW (including *Xba *I restriction enzyme sites) 5'-CTAGTCTAGAAACAGAGGCTAT TGTCCCC-3' and MUT-BW 5'- CTAGTCTAGAGCCATTTACCAAAGACTTTTTTTTTC -3' with MDA-MB-231 cDNA as a template. PCR was performed with *pfu *polymerase (SUN gene, Korea) methods. The resulting PCR fragments were cloned into the pGL3 vector by using *Xba *I restriction enzyme sites, creating the pGL3 CCR7 3'UTR WT and pGL3 CCR7 3'UTR MUT constructs. All PCR products were verified by DNA sequencing.

### Luciferase assay

Luciferase assays were performed in HEK-293 cells. HEK-293 cells were transfected with each of the plasmids (empty vector (EV), CCR7 3'UTR WT and CCR7 3'UTR MUT, as a let-7a binding site) together with synthetic let-7a oligonucleotides and negative control RNA in six-well plates. Forty-eight hours after transfection, the cells were harvested and lysed. Luciferase assays were performed on the lysates by using a luciferase assay kit (Promega). The luciferase activity was normalized to β-galactosidase.

### Northern blot analysis

Total RNA was isolated from human breast cancer cell lines by using TRIzol reagent (Invitrogen). RNA samples (15 μg each) were loaded on 15% acrylamide denaturing (urea) gels and then transferred onto nylon membranes. The hybridization with [γ-^32^P] ATP was performed at 37°C in a prehybridization solution (Clonetech) overnight. The membrane was washed at 37°C twice with 1 × SSC [0.3 *M *trisodium citrate and 3.0 *M *sodium chloride in high-purity dH_2_O, pH 7.0]. The oligonucleotide probes used were 5'-AACTATACAA CCTACTACCTCA-3' with a sequence complementary to the mature let-7a RNA. An oligonucleotide complementary to the U6 RNA, 5'-GCAGGGGCCATGCTAATCTTCTC TGTATCG-3', was used to normalize expression levels. Blots were stripped by boiling in 0.1% aqueous SDS in DEPC water for 1 minute and reprobed several times. As the loading control, tRNA stained with ethidium bromide was used.

### Cell-proliferation assay

Cell proliferation was measured with the MTT assay. MDA-MB-231 and MCF-7 cells were plated in 96-well culture plates (3 × 10^3 ^per well), followed by transfection of synthetic let-7a, CCR7 siRNA, anti-let-7a, and scRNA. After 48 hours of incubation, the MTT (0.5 mg/ml) (Sigma-Aldrich) was subsequently added to each well (200 μl/well). After 4 hours of additional incubation, the MTT solution was discarded; and 200 μl of DMSO (Amresco) was added, and the plate was shaken gently. The absorbance was measured on an enzyme-linked immunosorbent assay (ELISA) reader at a wavelength of 570 nm.

### Statistical analysis

Data are presented as mean ± SD from at least four to five images obtained from three independent experiments by using a microscope (×200), unless otherwise indicated. Statistical analysis was performed with one-way ANOVA by using Prism software and the Student *t *test for comparison between groups. Data were considered significant if *P *< 0.05.

## Results

### CCR7 and its ligand CCL21 were highly expressed in breast cancer cell lines and breast cancer patients

**Figure 1:**
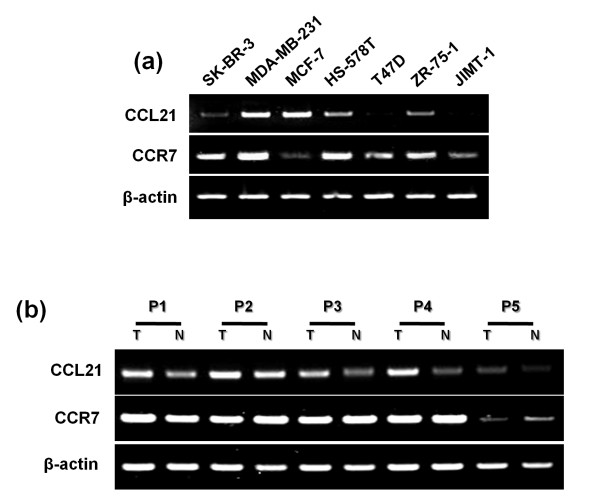
**Detection of mRNA expression levels of CCR7 and CCL21**. CCR7 and CCL21 mRNA expression levels were detected with RT-PCR analysis in seven breast cancer cell lines **(a) **and five breast cancer patient tissues **(b)**. β-actin was used as a normalization control.

First, the expression of mRNA for CCR7 and its ligands CCL19 and CCL21 was confirmed in breast cancer cell lines (Figure 1a) and in malignant and normal tissues from breast cancer patients (Figure 1b). CCR7 mRNA was highly expressed in most breast cancer cell lines and tissues from cancer patients. CCL19 expression was weakly detected in the cell lines and patient tissues (data not shown). However, CCL21 was more highly expressed in MDA-MB-231 and MCF-7 cell lines than in the other five breast cancer cell lines. A higher expression of CCL21 in malignant tissues than in their normal counterparts from breast cancer patients was discovered. Therefore, the results suggest that CCR7 signaling could be activated and affect cancer cells themselves by inducing strong expression of CCL21.

### Reverse correlation between the expression of let-7a and CCR7 in breast cancer cell lines

**Figure 2:**
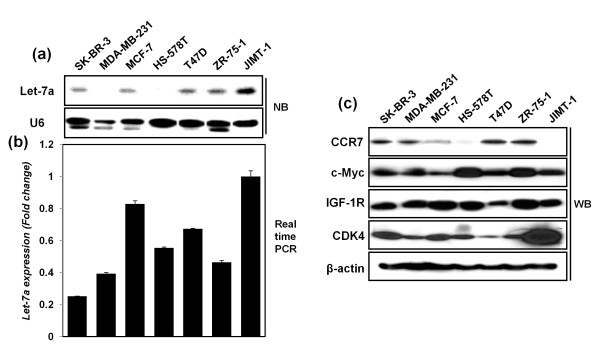
**Detection of basal expression levels of CCR7, IGF-1R, c-*Myc*, CDK-4, and let-7a in seven breast cancer cell lines**. **(a-b) **Let-7a expression levels in breast cancer cells, detected with Northern blot analysis (NB)(a) and real-time RT-PCR**(b)**. U6 and RNU6B were used as a normalization control. **(c) **Detection of protein expression levels of CCR7, IGF-1R, c-*Myc*, and CDK-4 by using Western blot analysis. β-actin was used as a normalization control.

Downregulation of let-7a expression was reported in breast cancer [19], and in the present study, the expression was determined with Northern blotting and quantitative RT-PCR analysis (Figure 2a, b). Comparatively, high and low let-7a expression was detected in MCF-7 and JIMT-1 cells and in MDA-MB-231 cells, respectively. To determine whether any correlation existed between the expression of let-7a and its target proteins in breast cancer cell lines, the expression levels of the predicted let-7a target proteins were examined, such as CCR7, IGF-1R, c-*Myc*, and CDK4 (Figure 2c). Among the target proteins, CCR7 showed a significant reverse correlation with let-7a expression, suggesting that CCR7 could be regulated by let-7a in breast cancer cells.

### Overexpression of let-7a decreases CCR7 expression as well as cell proliferation, invasion, and migration in MDA-MB-231 breast cancer cells

**Figure 3:**
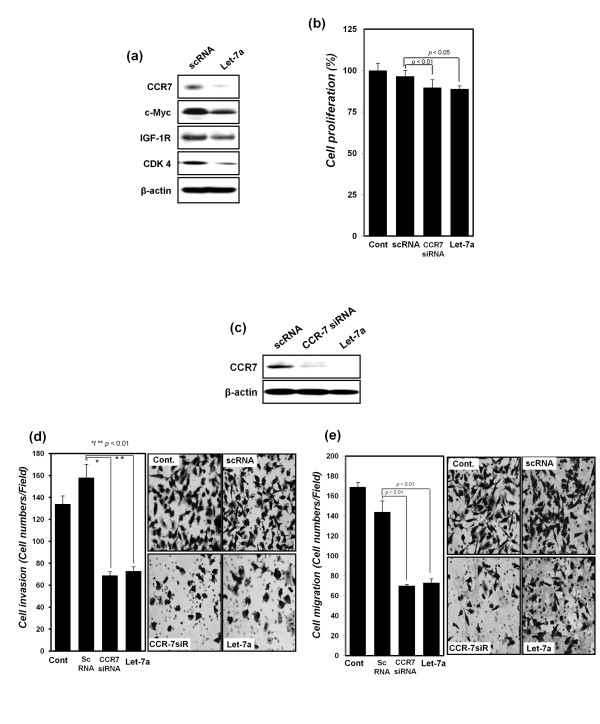
**Detection of changes in the expression of let-7a predicted target proteins and cell proliferation, cell migration, and invasion of MDA-MB-231 breast cancer cells after transfection with synthetic let-7a**. **(a) **After transfection with scRNA or synthetic let-7a, protein expression of CCR7, IGF-1R, c-*Myc*, and CDK-4 was detected with Western blot analysis. **(b) **Proliferation of MDA-MB-231 cells was assessed with MTT assay after transfection with scRNA, CCR7 siRNA, and synthetic let-7a, respectively. **(c) **Protein expression of CCR7 was detected with Western blot analysis after transfection with scRNA, CCR7 siRNA, and synthetic let-7a, respectively. β-actin was used as a normalization control. **(d) **Cell-migration assays and **(e) **invasion assays performed after transfection with scRNA, CCR7 siRNA, and synthetic let-7a, as described in Materials and Methods, presented as a histogram (left panel) with cell photos (right panel) All the experiments were performed in triplicate independently.

Synthetic let-7a was transfected into MDA-MB-231 cells, which express low levels of let-7a, and the change in the expression level of predicted CCR7 target proteins (Figure 3a) was evaluated. The expression of CCR7 and CDK4 was downregulated by let-7a more than that of IGF-1R and c-*Myc*. Recent studies have shown that both let-7a and CCR7 influence cell proliferation, as well as cancer cell invasion and migration [5,14,20]. To confirm the results, MDA-MB-231 cells were transfected with synthetic let-7a or CCR7 siRNA as a CCR7-silencing positive control (Figure 3b). According to the results, a small reduction in cell proliferation was detected by silencing CCR7 with both synthetic let-7a and CCR7 siRNA in transfected MDA-MB-231 cells. Moreover, both synthetic let-7a and CCR7 siRNA reduced cell invasion and migration by less than half of the reduction resulting from transfection with scRNA (Figure 3d, e). The results suggest that CCR7 silencing has an inhibitory effect on breast cancer cell proliferation, migration, and invasion, and overexpression of let-7a has the same effect as CCR7 silencing.

### Downregulation of let-7a increases cell invasion, migration, and proliferation in MCF-7 breast cancer cells

**Figure 4:**
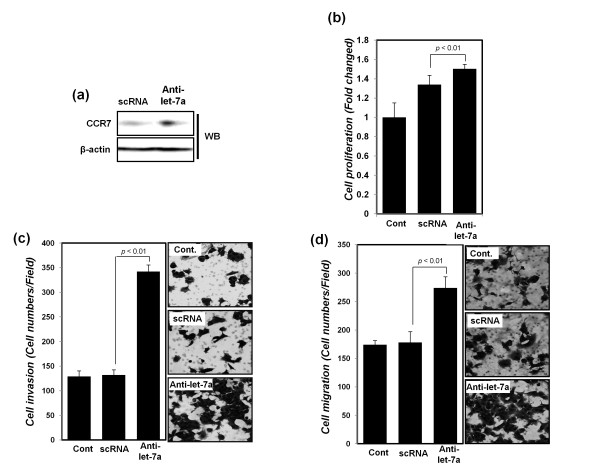
**Cell proliferation, cell migration, and invasion of MCF-7 breast cancer cell lines were increased after transfection with anti-let7a**. **(a) **Protein expression of CCR7 was detected with Western blot analysis after transfection with scRNA and anti-let-7a, respectively. β-actin was used as a normalization control. After transfection with scRNA or anti-let7a, **(b) **cell proliferation by MTT analysis, **(c) **cell-migration assays, and **(d) **invasion assays were performed, as described in Material and Methods, and the results of **(c) **and **(d) **presented as a histogram (left panel) with cell photos (right panel). All experiments were performed in triplicate independently.

Next, the inhibitory effect of let-7a was determined by using synthetic anti-let-7a oligo-nucleotides in MCF-7 breast cancer cells, which express a high level of let-7a and a low level of CCR7, with a high level of CCL21 expression. After transfection with synthetic anti-let-7a, the level of CCR7 expression increased (Figure 4a). The cell proliferation, migration, and invasion in MCF-7 cells also were assayed. As expected, transfection with anti-let-7a increased cell proliferation, invasion, and migration (Figure 4b-d). The results provided confirmation that CCR7 expression and the consequent breast cancer cell proliferation and motility are downregulated by let-7a.

### Let-7a regulation of cell migration and invasion is dependent on CCR7 and its ligand CCL21

**Figure 5:**
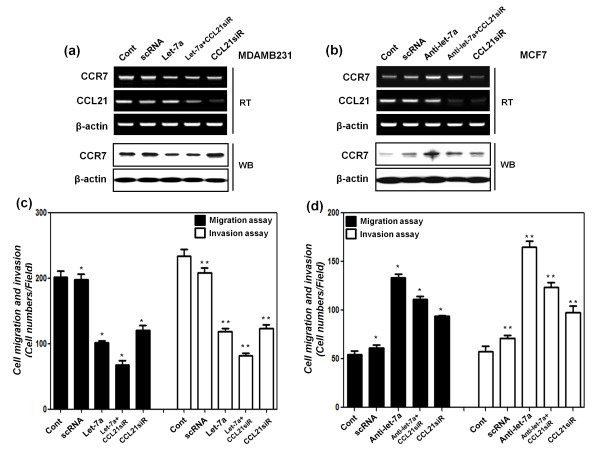
**Let-7a regulates cell migration and invasion of MDA-MB-231 and MCF-7 cell lines, dependent on CCR-7-CCL21 signaling**. **(a) **mRNA expression of CCL21 and CCR7, and protein expression of CCR7 were detected with RT-PCR and Western blot analysis after transfection with scRNA, synthetic let-7a, and CCL21 siRNA in MDA-MB-231 cells, respectively. β-actin was used as a normalization control. **(b) **mRNA expression of CCL21 and CCR7, and protein expression of CCR7 were detected with RT-PCR and Western blot analysis after transfection with scRNA, anti-let-7a, and CCL21 siRNA in MCF-7 cells, respectively. β-actin was used as a normalization control. **(c) **After transfection with scRNA, synthetic let-7a, and CCL21 siRNA into MDA-MB-231cells, cell-migration assays and cell-invasion assays were performed (*^, ^**P < 0.01 versus control group). **(d) **After transfection with scRNA, synthetic anti-let-7a, and CCL21 siRNA into McF-7 cells, cell-migration assays and cell-invasion assays were performed (*^, ^**P < 0.01 versus control group). The assays were described in Material and Methods, and the results of **(c) **and **(d) **are presented as a histogram. All experiments were performed in triplicate independently.

Additionally, the relation between let-7a and the CCL21-CCR7 signaling pathway was confirmed. First, CCL21-specific siRNA and synthetic let-7a were transfected into MDA-MB-231 cell lines expressing high levels of both CCL21 and CCR7 (Figure 5a and 5c). After CCL21 siRNA and synthetic let-7a transfections, downregulation of both CCL21 and CCR7 expression was shown via RT-PCR and Western blot analysis (Figure 5a). In addition, the effect of cell migration and invasion activity was confirmed through the cell migration and invasion assay, indicating that let-7a decreased MDA-MB-231 cell migration and invasion activity, dependent on CCR7 and CCL21 expression (Figure 5c).

Next, the anti-let-7a effect was also confirmed through the transfection of CCL21 siRNA and synthetic anti-let-7a in MCF-7 cell lines with a high expression of CCL21 and low expression of CCR7 (Figure 5b and 5d). After siRNA of CCL21 and synthetic anti-let-7a transfections, downregulation of CCL21 and overexpression of CCR7 expression was demonstrated by using RT-PCR and Western blot analysis (Figure 5b). Additionally, the effect of cell migration and invasion activity was confirmed by the cell migration and invasion assays, showing anti-let-7a increased MCF-7 cell migration and invasion activity, dependent on CCR7 and CCL21 expression (Figure 5d). These experiments showed that let-7a regulates CCR7 and cell motility, dependent on CCL21 expressions.

### Let-7a directly regulates CCR7 expression by binding with the 3'UTR of CCR7

**Figure 6:**
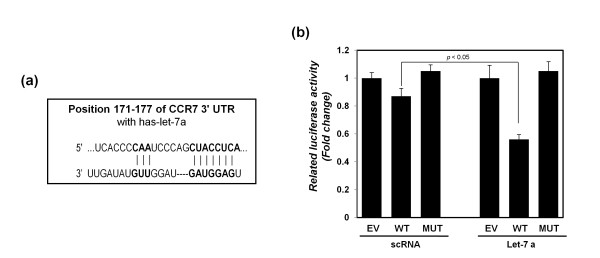
**Direct interaction between let-7a and 3'UTR of CCR7**. **(a) **Preparation of the construct with the let-7a binding site on the 3**'**UTR of CCR7, which was predicted by the TargetScan 4.1 database program. **(b) **Luciferase activity of CCR7 3'UTR luciferase reporters was detected after synthetic let-7a transfection. Wild-type (WT) and mutant type (MUT) of CCR7 3'UTR luciferase reporters were prepared as described in the Material and Methods section. Expression of β-galactosidase was used as a control for transfection normalization.

To elucidate the role of let-7a in CCR7 protein expression, the CCR7 3'UTR was prepared in the pGL3 control luciferase vector, and luciferase activity was evaluated after transfecting with synthetic let-7a (Figure 6). First, the let-7a binding site on CCR7 3'UTR was predicted by using the TargetScan microRNA-binding prediction program (Figure 6a). Next, the wild-type (WT) constructs containing let-7a binding CCR7 3'UTR and mutant type (MUT) constructs were prepared, in which the binding site was deleted. After transfection with scRNA or synthetic let-7a, the luciferase activity was analyzed (Figure 6b). Synthetic let-7a transfection had greater luciferase activity in the CCR7 3'UTR WT construct than in the CCR7 3'UTR MUT construct. The result suggests that let-7a directly regulates CCR7 protein expression through interaction with the 3'UTR of CCR7.

### Transfection of synthetic let-7a decreases *in vivo *breast cancer cell invasion in zebrafish embryo animal models

**Figure 7:**
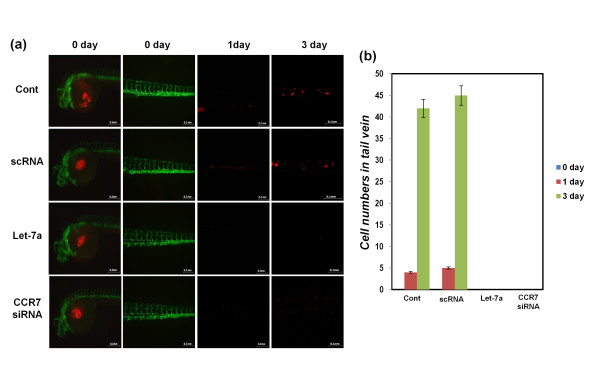
**Detection of the migration of breast cancer cells in zebrafish embryos as an *in vivo *animal model**. **(a, b) **RFP-labeled MDA-MB-231 cells were transfected with synthetic let-7a, CCR7 siRNA, and scRNA, respectively, and injected into the center of the yolk sac of transgenic zebrafish in which embryonic vessels are visualized with green fluorescence, as described in the Material and Methods section. After 1 day and 3 days of injections, RFP-labeled MDA-MB-231 cells were detected in GFP-labeled vessels by using a fluorescence microscope, and the results are presented as a photograph in **(a)**. The number of migrating cells was counted **(b)**. The data were derived from three replicated experiments. The scale bars are 200 μm and 50 μm in the last panels.

To analyze the *in vivo *effect of let-7a on cancer cell migration, zebrafish embryos having green fluorescent protein (GFP)-labeled blood vessels were prepared as an animal model. Red fluorescent protein (RFP)-labeled MDA-MB-231 cells were transfected with synthetic let-7a or CCR7 siRNA and injected into the abdomens of the zebrafish embryos. After each time point, the cells that migrated out into the blood vessels in the zebrafish embryos were detected (Figure 7a), and the zebrafish embryos containing the migrating cells were counted (Figure 7b and Tables 2 and 3). Notably, control MDA-MB-231 cells migrated into the trunk regions and resided in the vessel of the trunk and/or tail of the zebrafish embryos after injection of the cells into their abdomens. However, synthetic let-7a or CCR7 siRNA-transfected cells were not detected in the trunk or tail vessels of the zebrafish embryos. The zebrafish embryos in which the detected cells were migrating to the trunks or tail vessels also were counted (Table 2). When using scRNA-transfected cells, breast cancer cell migration was observed in nine of 21 zebrafish embryos, and no cell migration was observed in embryos by using synthetic let-7a or CCR7 siRNA-transfected cells. The migrated cells in the trunks or tail vessels also were counted (Table 3). Approximately 30% of control breast cancer cells were able to migrate out from the embryo abdomen, but none of the synthetic let-7a or CCR7 siRNA-transfected cells could do so. Collectively, the data suggest that the zebrafish embryo model can be used to monitor the migration of breast cancer cells in a living animal, and let-7a overexpression or CCR7 silencing could cause a significant reduction in breast cancer cell migration *in vivo*.

**Table 2:** The number of zebrafish embryos with migrating MDA-MB-231 (RFP) cells transfected with let-7a, CCR7 siRNA and scRNA, respectively

Transfection	Embryos containing migrating cells/Total embryos		
	
	0 (day)	1 (day)	3 (days)
**Control**	0/25	7/25 (28%)	9/23 (39%)

**RFP scRNA**	0/25	8/24 (33%)	9/21 (43%)

**RFP let-7a**	0/27	0/26 (0%)	0/24 (0%)

**RFP CCR7 siRNA**	0/24	0/24 (0%)	0/21 (0%)

**Table 3:** The number of migrated cells per tail vein of zebrafish embryos that had migrating MDA-MB-231 (RFP) cells transfected with let-7a, CCR7 siRNA and scRNA, respectively

Transfection	Cells in the tail vein/Total injected cells		
	
	0 day	1 day	3 day
**Control**	0/150 (0)	4/150 (3%)	42/150 (28%)

**RFP scRNA**	0/150 (0)	5/150 (3%)	45/150 (30%)

**RFP let-7a**	0/150 (0)	0/150 (0)	0/150 (0)

**RFP CCR7 siRNA**	0/150 (0)	0/150 (0)	0/150 (0)

### Reverse correlation between the expression of CCR7 and let-7a in breast cancer patients

**Figure 8:**
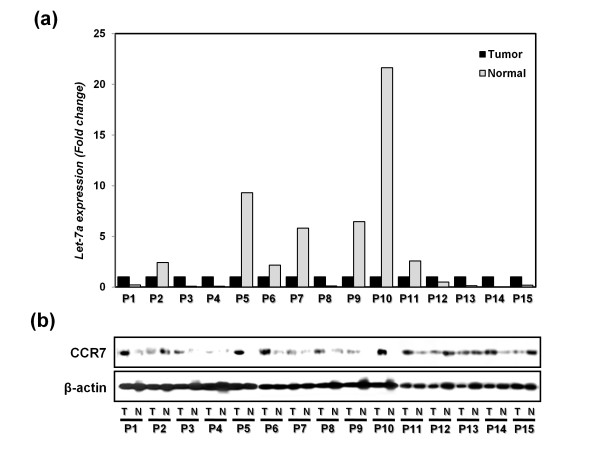
**Analysis of let-7a and CCR7 expression in tissues from 15 breast cancer patients (five infiltrating ductal cancers (P1, P3, P11-13), one metaplastic cancer matrix-producing type (P2), seven invasive ductal cancers (P4-10), one infiltrating cribriform cancer (P14), and one atypical medullary cancer (P15)] (Table 1)**. **(a) **Let-7a expression levels were determined by using real-time RT-PCR in malignant tissues (T) and their normal counterparts (N) from breast cancer patient tissues. RNU6B was used as a normalization control. **(b) **CCR7 expression in both malignant and normal tissues was detected by using Western blotting. β-actin was used a normalization control.

The expression of let-7a and CCR7 was examined in 15 breast cancer patients by quantitative RT-PCR analysis and Western blotting, respectively (Figure 8). In seven of 15 breast cancer patients, let-7a expression was more downregulated in malignant tissues than in normal counterpart tissues (Figure 8a). In contrast, CCR7 expression was higher in malignant tissues compared with normal counterpart tissues in 10 of 15 breast cancer patients (Figure 8b). A reverse correlation of expression was also found in tissues from six breast cancer patients.

**Table 1:** List of breast cancer patients

Number	Sex/Age	Pathologic diagnosis
1	F/54	Infiltrating ductal cancer
2	F/39	Metaplastic ca, matrix-producing type
3	F/60	Metaplastic ca, matrix-producing type
4	F/43	Invasive ductal cancer
5	F/63	Invasive ductal cancer
6	F/47	Invasive ductal cancer
7	F/46	Invasive ductal cancer
8	F/40	Invasive ductal cancer
9	F/54	Invasive ductal cancer
10	F/46	Invasive lobular cancer
11	F/52	Infiltrating ductal cancer
12	F/50	Infiltrating ductal cancer
13	F/51	Infiltrating ductal cancer
14	F/79	Infiltrating cribriform cancer
15	F/46	Atypical medullary cancer

## Discussion

The miRNA is highly conserved noncoding RNA that posttranscriptionally controls gene expression by degradation of target mRNA and/or by the inhibition of protein translation [15,21,22]. The miRNA contains an average of 22 nucleotides and plays important roles in a wide range of biologic processes, such as development, cell growth, cell proliferation, and death [23]. The deregulation of miRNA, frequently detected in human cancers, has been established as contributing to cancer progression and metastasis [24,25]. Additionally, miRNA has been identified as potential oncogenes or tumor suppressors [15,26].

Downregulation of let-7a was found in highly metastatic human breast cancer patient tissues [19], and in human breast cancer cells, such as MDA-MB-231 cells. To clarify the role of let-7a in metastasis, potential let-7a target genes were searched, specifically metastasis-related genes, and their protein expression was examined after synthetic let-7a treatment. Notably, a reverse correlation between levels of let-7a and CCR7 expression was found in both human breast cancer patient tissues and in cancer cell lines. CCR7 is known to play an important role in cancer metastasis [6]. We observed decreasing MDA-MB-231 cell migration and invasion when CCR7 expression was inhibited by synthetic let-7a. In contrast, silencing (inhibition) of let-7a resulted in increases in CCR7 expression, cell migration, and cell invasion in MCF-7 breast cancer cells, consistent with the results of CCR7 silencing by its specific siRNA. Therefore, we suggest that let-7a reduces breast cancer cell migration and invasion through the downregulation of CCR7 expression.

Normally, CCR7 activity is regulated by CCL19 and CCL21, which is secreted by cancer cells themselves and allows cancer cells to migrate to the lymphoid tissues. In the present study, CCL21 expression in breast cancer cell lines and in breast cancer patients was evaluated. As hypothesized, the expression levels of CCR7 and CCL21 were significantly increased in cancer cells and cancerous tissues from patients. In addition, we confirmed that CCR7 increased cell motility, dependent on CCL21 expression (Figure 5). The results suggest that CCL21 triggers the activation of CCR7 signaling through CCL21 secreted from cancer cells in an autocrine manner.

Based on our data, we can infer that let-7a regulates the translation of CCR7, which is believed to be the most common mechanism of miRNA targeting [27]. Let-7a targeting of CCR7 3'UTR was confirmed by using a luciferase reporter gene carrying the 3'UTR of CCR7 wild-type or a mutant type of the let-7a binding site. The data showed the 3'UTR of CCR7 was a direct target of let-7a. The effects of let-7a and CCR7 siRNA silencing on breast cancer cell migration *in vivo *were also confirmed by using a zebrafish embryo model. The studies showed that synthetic let-7a- or CCR7 siRNA-transfected cells exhibit reduced cell migration. The zebrafish embryo model was established to study cell migration *in vivo *because the RFP-labeled MDA-MB-231 cells could be detected easily in transparent zebrafish embryos containing GFP-labeled vessels. Normal MDA-MB-231 cells have the ability to migrate out into the vessel and move toward the tail, whereas cells in which CCR7 is silenced with synthetic let-7a or CCR7 siRNA lose this ability. Collectively, the results from the present study demonstrate let-7a suppresses metastasis through CCR7 target regulation and may be potentially useful as an antimetastatic agent in breast cancer.

## Conclusions

In the present study, we determined that let-7a suppressed breast cancer cell migration and invasion by downregulating CCR7 expression. Therefore, we suggest that targeting of CCL21-CCR7 signaling is a convincing approach for improving breast cancer therapy and the usefulness of let-7a as a direct regulator of this signaling. In addition, the strong association between the loss of let-7a expression and metastatic relapse suggests the potential of let-7a in prognostic stratification of breast cancer patients in addition to conventional clinical and pathologic staging markers.

## Abbreviations

3'UTR: 3'-untranslated region; CCR7: C-C chemokine receptor type 7; CCL19: C-C motif chemokine 19; CCL21: C-C motif chemokine 21; CDK-4: cyclin-dependent kinase-4; IGF-R: insulin-like growth factor receptor 1; MUT: mutant type; RT-PCR: RNA isolation and reverse transcription-polymerase chain reaction; scRNA: scrambled siRNA; siRNA: small interfering RNA; WT, wild type.

## Competing interests

The authors declare that they have no competing interests.

## Authors' contributions

SJK, JYS, and KDL contributed to concept design, established the RFP stable cell lines, carried out most of the molecular and functional studies, contributed to data analysis and interpretation, and drafted the manuscript. KHC contributed to concept design, data analysis and interpretation, manuscript writing, and funding. YKB contributed to the establishment of *in vivo *zebrafish migration experiments and the data analysis. KWS and SJN contributed to preparation of the breast cancer patient samples and patient information. All authors read and approved of the final manuscript.

## References

[B1] Jemal A Siegel R Ward E Hao Y Xu J Murray T Thun MJ Cancer statistics, 2008 CA Cancer J Clin 2008 58 71 96 10.3322/CA.2007.0010 18287387

[B2] Gonzalez-Angulo AM Morales-Vasquez F Hortobagyi GN Overview of resistance to systemic therapy in patients with breast cancer Adv Exp Med Biol 2007 608 1 22 10.1007/978-0-387-74039-3_1 17993229

[B3] Beauvillain C Cunin P Doni A Scotet M Jaillon S Loiry ML Magistrelli G Masternak K Chevailler A Delneste Y CCR7 is involved in the migration of neutrophils to lymph nodes Blood 2011 117 1196 204 21051556 10.1182/blood-2009-11-254490

[B4] Ben-Baruch A Organ selectivity in metastasis: regulation by chemokines and their receptors Clin Exp Metastasis 2008 25 345 56 10.1007/s10585-007-9097-3 17891505

[B5] Cunningham HD Shannon LA Calloway PA Fassold BC Dunwiddie I Vielhauer G Zhang M Vines CM Expression of the C-C chemokine receptor 7 mediates metastasis of breast cancer to the lymph nodes in mice Transl Oncol 2010 3 354 61 21151474 10.1593/tlo.10178 PMC3000460

[B6] Ben-Baruch A The multifaceted roles of chemokines in malignancy Cancer Metastasis Rev 2006 25 357 71 10.1007/s10555-006-9003-5 17016763

[B7] Weninger W von Andrian UH Chemokine regulation of naive T cell traffic in health and disease Semin Immunol 2003 15 257 70 10.1016/j.smim.2003.08.007 15001175

[B8] Takeuchi H Fujimoto A Tanaka M Yamano T Hsueh E Hoon DS CCL21 chemokine regulates chemokine receptor CCR7 bearing malignant melanoma cells Clin Cancer Res 2004 10 2351 8 10.1158/1078-0432.CCR-03-0195 15073111

[B9] Shields JD Emmett MS Dunn DB Joory KD Sage LM Rigby H Mortimer PS Orlando A Levick JR Bates DO Chemokine-mediated migration of melanoma cells towards lymphatics: a mechanism contributing to metastasis Oncogene 2007 26 2997 3005 10.1038/sj.onc.1210114 17130836

[B10] Reinhart BJ Slack FJ Basson M Pasquinelli AE Bettinger JC Rougvie AE Horvitz HR Ruvkun G The 21-nucleotide let-7 RNA regulates developmental timing in *Caenorhabditis elegans* Nature 2000 403 901 6 10.1038/35002607 10706289

[B11] Johnson SM Grosshans H Shingara J Byrom M Jarvis R Cheng A Labourier E Reinert KL Brown D Slack FJ RAS is regulated by the let-7 microRNA family Cell 2005 120 635 47 10.1016/j.cell.2005.01.014 15766527

[B12] Qian ZR Asa SL Siomi H Siomi MC Yoshimoto K Yamada S Wang EL Rahman MM Inoue H Itakura M Kudo E Sano T Overexpression of HMGA2 relates to reduction of the let-7 and its relationship to clinicopathological features in pituitary adenomas Mod Pathol 2009 22 431 41 10.1038/modpathol.2008.202 19136928

[B13] Lee YS Dutta A The tumor suppressor microRNA let-7 represses the HMGA2 oncogene Genes Dev 2007 21 1025 30 10.1101/gad.1540407 17437991 PMC1855228

[B14] Akao Y Nakagawa Y Naoe T let-7 microRNA functions as a potential growth suppressor in human colon cancer cells Biol Pharm Bull 2006 29 903 6 10.1248/bpb.29.903 16651716

[B15] Zhang H Li Y Lai M The microRNA network and tumor metastasis Oncogene 2010 29 937 48 10.1038/onc.2009.406 19935707

[B16] Zhu YM Zhong ZX Liu ZM Relationship between let-7a and gastric mucosa cancerization and its significance World J Gastroenterol 2010 16 3325 9 10.3748/wjg.v16.i26.3325 20614490 PMC2900726

[B17] Long XB Sun GB Hu S Liang GT Wang N Zhang XH Cao PP Zhen HT Cui YH Liu Z Let-7a microRNA functions as a potential tumor suppressor in human laryngeal cancer Oncol Rep 2009 22 1189 95 19787239 10.3892/or_00000554

[B18] Kim SJ Choi IJ Cheong TC Lee SJ Lotan R Park SH Chun KH Galectin-3 increases gastric cancer cell motility by up-regulating fascin-1 expression Gastroenterology 2010 138 1035 45 e1031-2 10.1053/j.gastro.2009.09.061 19818782

[B19] Iorio MV Ferracin M Liu CG Veronese A Spizzo R Sabbioni S Magri E Pedriali M Fabbri M Campiglio M Ménard S Palazzo JP Rosenberg A Musiani P Volinia S Nenci I Calin GA Querzoli P Negrini M Croce CM MicroRNA gene expression deregulation in human breast cancer Cancer Res 2005 65 7065 70 10.1158/0008-5472.CAN-05-1783 16103053

[B20] Yu F Yao H Zhu P Zhang X Pan Q Gong C Huang Y Hu X Su F Lieberman J Song E let-7 regulates self renewal and tumorigenicity of breast cancer cells Cell 2007 131 1109 23 10.1016/j.cell.2007.10.054 18083101

[B21] Zhang H Li Y Lai M The microRNA network and tumor metastasis Oncogene 2010 29 937 48 10.1038/onc.2009.406 19935707

[B22] Garzon R Marcucci G Croce CM Targeting microRNAs in cancer: rationale, strategies and challenges Nat Rev Drug Discov 2010 9 775 89 10.1038/nrd3179 20885409 PMC3904431

[B23] Ambros V microRNAs: tiny regulators with great potential Cell 2001 107 823 6 10.1016/S0092-8674(01)00616-X 11779458

[B24] Yu Z Baserga R Chen L Wang C Lisanti MP Pestell RG microRNA, cell cycle, and human breast cancer Am J Pathol 2010 176 1058 64 10.2353/ajpath.2010.090664 20075198 PMC2832125

[B25] Cowland JB Hother C Gronbaek K MicroRNAs and cancer APMIS 2007 115 1090 106 10.1111/j.1600-0463.2007.apm_775.xml.x 18042145

[B26] Zhang B Pan X Cobb GP Anderson TA microRNAs as oncogenes and tumor suppressors Dev Biol 2007 302 1 12 10.1016/j.ydbio.2006.08.028 16989803

[B27] Lee RC Ambros V An extensive class of small RNAs in *Caenorhabditis elegans* Science 2001 294 862 4 10.1126/science.1065329 11679672

